# New-Onset Psoriatic Arthritis under Biologics in Psoriasis Patients: An Increasing Challenge?

**DOI:** 10.3390/biomedicines9101482

**Published:** 2021-10-15

**Authors:** Matteo Megna, Sonia Sofia Ocampo-Garza, Luca Potestio, Giuseppina Fontanella, Lucia Gallo, Sara Cacciapuoti, Angelo Ruggiero, Gabriella Fabbrocini

**Affiliations:** 1 Section of Dermatology, Department of Clinical Medicine and Surgery, University of Naples Federico II, Via Pansini 5, 80131 Napoli, Italy; mat24@libero.it (M.M.); dra.soniaocampo@gmail.com (S.S.O.-G.); potestioluca@gmail.com (L.P.); giusifontanella@gmail.com (G.F.); luciagallo890@gmail.com (L.G.); sara.cacciapuoti@libero.it (S.C.); angeloruggiero1993@libero.it (A.R.); 2 Dermatology Department, Hospital Universitario “Dr. José Eleuterio González”, Universidad Autónoma de Nuevo León, Monterrey 64460, Mexico

**Keywords:** psoriasis, psoriatic arthritis, biologic therapies, paradoxical reaction, anti-TNF, anti-IL12/23, anti-IL17, anti-IL23

## Abstract

Psoriasis and psoriatic arthritis (PsA) development is sustained by tumor necrosis factor (TNF)α, interleukin (IL)17, and IL23; hence, biologics targeting those cytokines represent useful therapeutic weapons for both conditions. Nevertheless, biologics strongly reduce PsA risk; several studies reported the possibility of new-onset PsA during biologic therapy for psoriasis. The aim of this 1-year prospective study is to evaluate the prevalence of paradoxical PsA in psoriasis patients under biologic therapy and review the existing literature. For each patient, age, sex, psoriasis duration, psoriasis severity, comorbidities, and previous and current psoriasis treatments were collected, and each subject was screened for PsA using the Early ARthritis for Psoriatic patient (EARP) questionnaire every 3 months for 1 year. New-onset PsA was diagnosed in 10 (8.5%) out of 118 patients (three male, 30.0%; mean age 44.5 years) involving every different biologic class (anti-TNF, anti-IL12/23, anti-IL17, and anti-IL23). No significant risk factor for new-onset PsA was identified; no significant difference was found comparing patients who developed PsA and subjects who did not develop PsA regarding psoriasis severity, past/current therapies, and comorbidities. Clinicians must keep in mind the possibility of PsA onset also in patients undergoing biologics so that PsA screening should be strongly recommended at each follow-up.

## 1. Introduction

### 1.1. Psoriasis

Psoriasis is a chronic inflammatory disease affecting about 1–3% of adults and 0.1–0.3% of children in Western Europe [1]. It is commonly characterized by the presence of well-delineated papulosquamous plaques surrounded by normal skin and covered by silvery scales [2,3].

Plaque psoriasis is the most common clinical presentation, accounting for more than 80% of cases [4].

The pathogenesis of psoriasis is not completely understood [5], and both genetics and immune factors are involved [6]. Psoriatic disease is not only limited to the skin; indeed, several comorbidities may be associated with psoriasis such as cardiovascular disease, inflammatory bowel disease, nonalcoholic fatty liver disease, obesity, diabetes mellitus, and psoriatic arthritis (PsA) [7].

In this context, the development of target therapies selectively blocking cytokines involved in psoriatic disease pathogenesis such as biologics has revolutionized both psoriasis and PsA treatment [8].

### 1.2. Psoriatic Arthritis

PsA is a chronic, inflammatory musculoskeletal disease with different clinical subtypes, which may change and overlap over time, as described by Moll and Wright [9,10] in 1973.

PsA may be a severe disease that severely affects quality of life, deeply impacting on daily life and limiting the functional movement of the involved joints [11].

Non-musculoskeletal manifestations can be associated with PsA such as skin and nails involvement, inflammatory bowel disease, and uveitis, often forcing a multidisciplinary approach [12].

Nevertheless, only 15% of patients show PsA before psoriasis [13], with about 30% of patients with psoriasis developing articular involvement about 10 years after the cutaneous one [14,15]. PsA is still a diagnostic challenge [16]. Indeed, the average diagnostic delay for PsA is 5 years [17] with about 15% of psoriatic patients having undiagnosed PsA [18].

Diagnostic delay is very important, since it has been shown that within 2 years from the onset of the disease, almost 50% of patients develop bone erosions [14] and that a diagnostic delay of 6 months represents a risk factor for radiographic progression and for long-term functional impairment [19].

### 1.3. Disease Pathogenesis and Biologic Therapies in Psoriasis and Psoriatic Arthritis

In the context of psoriatic disease, the pro-inflammatory cytokines that sustain both psoriasis and PsA development are represented by tumor necrosis factor (TNF)α, interleukin (IL)17, and IL23; hence, biologics and small molecules targeting those cytokines represent useful therapeutic weapons for both conditions at the same time [20].

For all these reasons, anti-TNFα, ustekinumab, anti-IL17, and anti-IL23 are expected to be effective for both conditions [20,21]. Indeed, they are all approved for PsA with brodalumab, risankizumab, and tildrakizumab awaiting FDA approval. However, several studies reported the possibility of new-onset of PsA during biologic therapies in psoriatic patients with two studies highlighting the concept of paradoxical PsA [22,23,24].

### 1.4. Paradoxical Reaction to Biologic Therapies

Paradoxical reaction is defined as the occurrence, during treatment with biologics, of a disease that is usually responsive to this class of drug [25].

Psoriasiform reaction, PsA, hidradenitis suppurativa, uveitis, inflammatory bowel disease, pyoderma gangrenosum, granulomatous reaction, and vasculitis are the most reported paradoxical reactions during biological treatment for different conditions such as psoriasis, Chron’s disease, and arthritis [26].

Up until now, there are only a few studies about the development of PsA in patients treated with biologic drugs, and data are limited to case reports and very limited real-world experiences.

### 1.5. Objective of the Study

The aim of this 1-year prospective study is to evaluate the prevalence of new-onset PsA in psoriasis patients under existing biologic therapy approved for psoriasis and reviewing the existing literature.

## 2. Materials and Methods

### 2.1. Setting and Study Population

A prospective study was carried out enrolling moderate-to-severe psoriatic patients under biologic treatment for their disease, attending the Psoriasis Care Centre of Dermatology at the University Federico II of Naples from March 2019 to April 2021.

Inclusion criteria were diagnosis of moderate-to-severe plaque psoriasis by a dermatologist, psoriasis duration >1 year, and treatment with biologic drugs approved for psoriasis (anti-TNFα: etanercept, adalimumab, certolizumab, infliximab; anti-IL12/23: ustekinumab; anti-IL17: secukinumab, ixekizumab, brodalumab; and anti-IL23: risankizumab, guselkumab, tildrakizumab) for at least 3 months.

Exclusion criteria were previous diagnosis of PsA or referred arthralgia, swollen joints, morning stiffness, and/or muscle and tendon pain before starting biologic therapy.

For each patient, the following data were collected: age, sex, psoriasis duration, psoriasis severity through Body Surface Area (BSA) and Psoriasis Activity Severity Index (PASI), comorbidities, and previous and current psoriasis treatments. All collected data are reported in Table 1.

Each patient performed a follow-up visit every 3 months for at least 1 year, accounting for a total of at least 5 visits for every patient. During each follow-up, psoriasis severity was evaluated with PASI and BSA, and routine blood tests were performed (erythrocyte sedimentation rate, rheumatoid factor, antibodies to cyclic citrullinated peptides, complete blood count, basic metabolic, lipid, liver, and renal panel); moreover, during each visit, the patients were screened for PsA using the Early ARthritis for Psoriatic patients (EARP) questionnaire [27].

Rheumatological referral with clinical examination and imaging (X-ray, ultrasound, and/or magnetic resonance imaging) was performed when the EARP score resulted > 3, suggesting the suspect of PsA onset.

The present study was conducted respecting the Declaration of Helsinki, and all patients signed an informed consent before starting the study.

### 2.2. Literature Review

As regards the review of the literature, a search of the Pubmed, Embase, and Cochrane Skin databases was performed (until 25 July 2021) using the following research terms: “paradoxical psoriatic arthritis”, “biologic drug”, “psoriatic arthritis”, “etanercept”, “adalimumab”, “certolizumab”, “infliximab”, “ustekinumab”, “secukinumab”, “ixekizumab”, “brodalumab”, “risankizumab”, “guselkumab”, and “tildrakizumab”. Articles regarding non-new onset of PsA during biologic treatments for psoriatic disease were excluded. Thus, the research was refined by reviewing the abstracts and texts of collected articles. English, Spanish, or French language articles have been considered. A total of 12 articles were selected for the evaluation in the present review.

### 2.3. Statistical Analysis

Descriptive statistics were performed for each demographic and clinical variable. Data were presented as mean ± standard deviation in case of continuous variables and as number and proportion of patients for categorical ones. Student’s *t*-test and Chi-square test were used to determine the statistical significance of the differences between the group of patients who developed PsA vs. the group of patients who did not experience a new onset of PsA during biologic therapies. In particular, the Chi-square test was used to assess the statistically significance of the differences between categorical variables and Student’s t-test was used for the continuous ones. *p*-values < 0.05 were considered to be statistically significant. All statistical analyses were performed using GraphPad Prism 4.0 (GraphPad Software Inc., La Jolla, CA, USA).

## 3. Results

### 3.1. Study Population

#### 3.1.1. Study Population: General Details

In our study, a total of 198 patients with plaque psoriasis without PsA were screened following the proposed inclusion and exclusion criteria. Among these, 131 (68.9%) patients respected all the inclusion and exclusion criteria. However, 12/131 (9.2%) patients did not complete the study: seven subjects had to switch the biologic treatment before 1 year follow-up due to lack of efficacy on psoriasis lesions and five patients were lost to follow-ups.

Finally, 118 subjects (76 male, 64.4%; 42 female, 35.6%; mean age 48.4 ± 12.1 years, range 9–80 years) completed the study (Table 1). Clinical examination revealed mean PASI of 2.2 ± 6.2 and mean BSA of 2.6 ± 5.6. The study population showed a considerable history of disease and related treatment. Indeed, the mean duration of psoriasis was 18.42 ± 12.4 years with an average duration of biologic treatment being 36.1 ± 26.4 months.

#### 3.1.2. Comorbidities

Half of the patients (59, 50.0%) reported at least one comorbidity. The most common comorbidity was dyslipidemia (24, 20.3%), followed by hypertension (16, 13.6%), obesity (15, 12.7%), and diabetes mellitus (12, 10.2%). The remaining comorbidities and other clinical data are reported in Table 1.

#### 3.1.3. Previous Treatments

All of the patients had been previously treated with at least one conventional systemic drug; in particular, cyclosporine (47, 39.8%) and methotrexate (40, 33.9%) resulted from the most commonly employed ones.

The majority of patients were bionaive (84, 71.2%). As regards bioexperienced subjects, 23/34 patients (67.6%) failed one biologic treatment, while nine (26.5%) and two (5.9%) subjects had been previously treated with two or more than two biologics, respectively. Anti-TNFα were the most frequently previously administered biologic treatments (26, 22.0%), followed by anti-IL17 (14, 11.9%), anti-IL12/23 (9, 7.6%), and anti-IL23 (1, 0.8%). Detailed data regarding each biologic are reported in Table 1.

#### 3.1.4. Current Treatments

As regards current therapy, ustekinumab was the most common ongoing treatment (27, 22.9%) followed by adalimumab (25, 21.2%), ixekizumab (25, 21.2%), secukinumab (13, 11.0%), guselkumab (11, 9.3%), brodalumab (6, 5.1%), tildrakizumab (4, 3.4%), etanercept (4, 3.4%), certolizumab (2, 1.7%), and risankizumab (1, 0.9%) (Table 1).

### 3.2. New-Onset PsA

#### 3.2.1. PsA Diagnosis

During our study, 15 (12.7%) out of 118 patients with plaque psoriasis on biologic treatment revealed symptoms suggestive of PsA and/or showed an EARP score > 3 during 1-year follow-up. These subjects were all referred to a rheumatologist who confirmed PsA diagnosis in only 10/15 (66.7%), whereas in the remaining five cases, a diagnosis of arthrosis, fibromyalgia, or arthralgia was performed. In each case, the rheumatologist performed a final diagnosis after clinical examination and at least one instrumental investigation preferring ultrasound for peripheral skeleton and magnetic resonance for axial involvement.

Thus, new-onset PsA (developed nevertheless the use of a biologic drug active for both psoriasis and PsA) was finally diagnosed in 10 (8.5%) out of 118 patients (three male, 30.0%; seven female, 70%; mean age 44.5 years, range 27–73 years) during 1 year follow-up under biologic therapy. Interestingly, these patients showed a very limited skin involvement, presenting a mean PASI of 1.8 ± 2.9 and a mean BSA of 4.6 ± 8.7 (Table 1). Clinical data and features of patients who develop or who do not develop PsA during biologic therapy at 1 year follow-up are reported in Table 1. Details of patients who developed new-onset PsA as well as their treatment have been reported in Table 2.

#### 3.2.2. PsA Clinical Subtypes, Severity, and Treatment

Among the patients with new-onset PsA, seven patients (70.0%) developed a peripheral articular involvement, two (20.0%) showed an axial articular disease, whereas one patient (10.0%) manifested both a peripheral and axial damage.

Concerning the peripheral form of PsA, four (57.1%) patients developed oligoarthritis, one (14.3%) developed entesitis, one developed dactylitis (14.3%), and one (14.3%) developed dactylitis and enthesitis.

Concerning the severity of PsA, mild and moderate forms prevailed (8,80%) with severe disease being assessed in only two cases (20%) (Table 2).

As regards the 10 patients who developed new onset PsA, in only three cases (30%), it was necessary to switch the biologic drug, while the majority (7, 70%) performed a combined therapy with ongoing biologic and a new drug; details are shown in Table 2.

#### 3.2.3. New-Onset PsA and Ongoing Biologic Treatments

The most common ongoing biologic treatments in the new-onset PsA group of subjects were adalimumab (two, 20.0%), ustekinumab (two, 20.0%) and secukinumab (two, 20.0%), followed by etanercept (one, 10.0%), ixekizumab (one, 10.0%), brodalumab (one, 10.0%), and risankizumab (one, 10.0%). The main biologic treatment duration in patients who developed PsA was 26.1 ± 21.6 months. In particular, patients undergoing biologic therapies with adalimumab, ustekinumab, secukinumab, etanercept, ixekizumab, brodalumab, and risankizumab had been treated for 15 ± 4.2, 39 ± 29.7, 40 ± 11.3, 56, 12, 3, and 2 months, respectively. Statistically significant differences comparing the current biologic treatment in the group of patients that developed PsA vs. the group of subjects who did not experience the PsA onset during biologic treatment regarding type and treatment duration were not found.

Half of the patients who developed PsA during biologic therapy were biologic naïve (5, 50.0%). Instead, the remaining had been treated with one, two, or three different biologic drugs respectively in two, two, and three patients.

#### 3.2.4. Comorbidities and PASI in Patients Who Developed PsA

Interestingly, the majority of patients with new-onset PsA (6/10, 60.0%) did not have comorbidity, whereas the main comorbidities present in the remaining four subjects were dyslipidemia, obesity, and hypertension.

Among patients that did not develop PsA during biologic therapy (108/118, 91.5%; M/F = 73/35), the mean PASI revealed was 2.2 ± 6.5 with a mean BSA of 2.4 ± 5.2 (Table 1).

#### 3.2.5. Patients Who Developed New-Onset PsA vs. Patients Who Did Not Develop PsA

No statistically significant difference was observed between new-onset PsA group and non-PsA group as regards ongoing treatments, nor was a significant trend between one drug or class of biologics and new-onset PsA risk observed.

Moreover, no cases of new-onset PsA were reported in patients treated with certolizumab, guselkumab, and tildrakizumab; however, these data did not approach statistical significance when compared to other treatments and were related to the very small sample size of patients that performed those treatments with respect to the others. Patients that experienced the development of PsA were compared to patients that did not develop PsA at 1 year follow-up for all the investigated outcomes. No statistically significant differences were found comparing sex, mean age, mean duration of psoriasis, BSA, PASI, comorbidities, and time and type of both previous and current treatment for the two groups.

## 4. Discussion

Psoriasis and PsA are both debilitant pathologies that require a multidisciplinary approach; an integrated dermatologic and rheumatologic examination allows the best therapy outcome in PsA patients [28]. As regards psoriatic disease pathogenesis, the pro-inflammatory cytokines sustaining psoriasis development (TNFα, IL17, and IL23) have been found to play a key role in PsA pathogenesis, too [20].

The management of PsA includes both pharmacological and non-pharmacological approaches with the aim of disease remission or low disease activity and is connected to the severity of PsA [12].

In this context, biologics are the only drugs that have shown their effectiveness in reducing and/or blocking the articular damage, thus preventing PsA progression [29,30,31] where traditional systemic therapies, including cyclosporine or methotrexate, have been shown to be not effective [32,33].

Patients on biologic drugs for psoriasis are expected to have a lower risk of PsA development, since they are acting on shared pro-inflammatory targets. However, there are still several limitations in the biologic treatment of psoriasis such as primary or secondary inefficacy, auto-antibodies formation, and the possibility of not preventing disease progression [33].

Indeed, different studies [22,23,24,34] and real-life experiences [35,36,37,38,39,40,41,42] showed the possibility of PsA onset in patients treated with biologics for psoriasis in 4.5–9.4% of cases [22,24]. The present study aims to evaluate the incidence of new-onset PsA in patients with plaque psoriasis on anti-TNFα (adalimumab, certolizumab, etanercept, infliximab), anti-IL12/23 (ustekinumab), anti-IL23 (risankizumab, tildrakizumab, guselkumab) or anti-IL17 (brodalumab, ixekizumab, secukinumab) therapy attending our Dermatology Unit and undergoing at least 1-year follow-up.

Our results showed that 8.5% (10 out of 118) of psoriasis patients under biologic therapy for plaque psoriasis developed new-onset PsA during one-year follow-up despite performing a treatment that would have been efficacious also for joint involvement. We did not observe a predilection for a particular class of biologic agents. Indeed, in our study population, we observed cases of emerging PsA in anti-TNFα, anti-IL23, 17, and 12/23 treated patients without any statistical difference. Particularly, three (30.0%) subjects were in treatment with anti-TNFα, four (40.0%) with anti-IL17, one (10.0%) with anti-IL23, and two (20.0%) with anti-IL12/23. Again, no significant difference was found between patients who develop PsA and patients who do not develop PsA as regards age, sex, psoriasis severity, psoriasis duration, and previous or current treatments. Interestingly, also, comorbidities did not significantly differ in these two groups, although it is well known in the literature that obesity and uveitis represent two major risk factors for PsA [43,44].

These results underline the possibility of PsA development also in patients under biologics that are already approved for PsA treatment (anti-TNFα, ustekinumab, anti-IL17 such as secukinumab and ixekizumab, and anti-IL23 such as guselkumab) or targeting cytokines involved in PsA pathogenesis (IL17 and IL23) such as brodalumab, risankizumab, and tildrakizumab, which are awaiting FDA approval for PsA.

After the diagnosis of PsA, a new therapy was added to current treatment in seven patients, whereas a new biologic drug was prescribed in the remaining cases (Table 2).

Even if it is well known that the TNF-α/IL-23/IL-17 axis is involved in the pathogenesis of psoriasis and PsA [45,46], it is not so easy to compare the efficacy of a selected biologic drug for skin and joints.

Indeed, the American College of Rheumatology core set (ACR) [47] and PASI response [48] are different assessment tools, so a direct comparation between the therapeutic effects of biologics in psoriasis and PsA is not possible. Clinical trials showed that anti-IL17 and anti-IL23 are more effective for psoriasis than anti-TNFα and ustekinumab, whereas they failed to demonstrate anti-IL17′s superiority vs. anti-TNFα agents for PsA; so anti-TNFα is still considered the mainstay treatment for PsA [45]. However, head-to-head studies (anti-IL17 vs. anti-TNFα) for patients with both psoriasis and PsA showed that ixekizumab was superior to adalimumab in the achievement of simultaneous improvement of joint and skin disease (combined ACR50 and PASI100) [49].

To our knowledge, studies [22,23,24] investigating PsA onset during psoriasis treatment with biologics are very limited (three existing studies).

Napolitano et al. [22] evidenced the possibility of PsA development during treatment with adalimumab, etanercept, infliximab, and/or ustekinumab for plaque psoriasis. In their retrospective study, 22 out of 327 patients (6.7%) developed PsA during biologic treatment in a 5-year retrospective study. Our study reported a slightly higher percentage (8.5%) of new-onset PsA, thus analyzing a wider study population (also patients under anti-IL17 or IL-23 had been considered). Hence, our results showed that PsA may occur in patient treated with anti-IL17 and IL-23 too.

Moreover, in both studies, the percentage of bionaïve patients in subjects that developed PsA was similar (12/22, 54,5% in Napolitano et al. vs. 5/10, 50%). The mean age of patients was similar as well (49.3 in their cohort vs. 48.4 in ours).

Regarding the pathogenesis of new-onset PsA, Napolitano et al. [22] suggested a relationship between the occurrence of PsA and the severity of psoriasis (in their study, 63.6% of patients who developed PsA had PASI > 10 with mean PASI of 26.8). Otherwise, this relationship was not confirmed in our study (mean PASI score of 1.8 ± 2.9), suggesting that psoriasis severity is not necessarily connected to the possibility of PsA development or at least that it does not constitute the only factor.

Despite being slightly higher than the results reported by Napolitano et al. [22], the percentage of new-onset PsA found in our study is slightly lower than those reported by Van Muijen et al. [23], who showed that 32 (9.4%) patients developed PsA during biologic treatment (adalimumab, etanercept, infliximab, ustekinumab, secukinumab) collecting data from the BioCAPTURE database from 1 May 2005 until 1 May 2018. However, their inclusion criteria were different from our study and from those of Napolitano et al. [22] concerning the phenotype of psoriasis. In fact, Van Muijen et al. [23] also included patients with guttate, pustular, and erythrodermic psoriasis, recording the localization and types (scalp lesions, nail psoriasis, inverse psoriasis, and palmoplantar psoriasis), whereas our study and Napolitano et al. [22] only considered plaque psoriasis, the only type of psoriasis for which biologics are approved. Moreover, Van Muijen et al. [23] reported a trend toward significance for a lower risk of PsA onset in patients with inverse psoriasis (OR 0.61, 95% CI 0.36–1.05, *p*-value 0.07), non-confirming literature data that reported an increased risk of PsA development in patients with scalp, nail, and inverse psoriasis [39].

Furthermore, Van Muijen et al. [23] analyzing all the variables of their cohort of patients reported that the male gender was the only significant variable associated with a lower risk of developing PsA (OR 0.58, 95% CI 0.34–0.98, *p*-value 0.04). According to them, in our study, there is a female predominance in patients who developed PsA (seven out of 10 patients, 70%).

On the other hand, this difference is not confirmed by Napolitano et al. [22], who reported that 15/22 (68.2%) patients who developed new-onset PsA were male.

Regarding the severity of psoriasis at the moment of PsA detection, the mean PASI revealed was 6.6 ± 6.6 with 53.8% of patients having a PASI < 5. This result is supported by the findings of our study (mean PASI 1.8 ± 2.9). Conversely, these data are not confirmed by those of Napolitano et al. (mean PASI 18.7 ± 12.1), proposing that psoriasis severity does not seem to play a key role in PsA onset.

However, the results of our study, those of Napolitano et al. [22], and Van Muijen et al. [23] completely agree that oligoarthritis is the most common clinical pattern of new-onset PsA, being found in 4/10 (40%), 7/22 (31.8%), and 19/32 (59.4%) of subjects who developed PsA in our study, in Napolitano et al. [22], and in the Van Muijen et al. [23] cohort, respectively, highlighting that the peripheral form of PsA seems to be the main phenotype of PsA developed during biologic treatment for psoriasis. These results suggest that the clinical features of new-onset of PsA during biologic treatment are similar to those of PsA with a prevalence of peripheral forms and a low percentage of axial ones (7–32%) [13].

A retrospective analysis involving patients treated with ustekinumab for psoriasis [24] reported that eight out of 179 patients (4.5%) developed a new-onset of PsA during the treatment. This study showed a male/female ratio of 3/1 with a mean PASI of 12.9 ± 5.1. Patients who developed PsA had a significantly lower body mass index (BMI) (21.2 ± 1.9 vs. 23.7 ± 3.3, *p* < 0.05). Moreover, half of the patients that developed PsA had smoke habits, and a nail involvement was found in five cases (63%).

In our study, of 27 patients treated with ustekinumab, two (7.4%) developed new-onset PsA (100% female). Particularly, all our patients that developed PsA were female, non-confirming the male prevalence of Asahina et al. (6/8, 75.0%). Moreover, the mean age of their patients who experienced the onset of PsA during biologic treatment was more than twice our patients’ age (64.1 ± 18.8 vs. 28.5 ± 2.1 years), and the mean duration of disease was higher as well (11.0 ± 11.7 vs. 8.5 ± 2.1 years). Finally, the psoriasis severity shown in their study was not confirmed in ours (PASI 12.9 ± 5.1 vs. 0 ± 0), non-highlighting a correlation with the possibility of PsA onset.

Studies reporting the development of new-onset PsA in patients with moderate-to-severe psoriasis treated with biologic therapy are summarized in Table 3.

Compared to the other studies, the incidence of PsA in our analysis seems to be higher than in other studies. This fact could be linked to the high specify of our department, which is accessed by patients with a diagnosis of difficult-to-treat psoriasis. However, our results are similar to those of Gisondi et al. (8.1%) and lower than those of Van Muijen et al. (9.4%), confirming the high reliability of our analysis.

In a cross-sectional study, investigating the prevalence of PsA in patients with severe psoriasis, Haaron et al. [34] reported the new diagnosis of PsA in 29 out of 100 patients. Among these, 11 patients (38.9%) were under biologic treatment (anti-TNFα or anti-IL12/23) at the moment of the diagnosis. However, the investigation about the development of PsA during biologic treatment was not the aim of their study, so detailed data on this topic are not reported in their study; hence, this study has not reported in Table 3.

In addition, some case reports show the onset of paradoxical PsA during treatment with biologic therapy for psoriasis [35,36,37,38,39,40,41] (see Table 3).

Finally, a survey [42] administered to 988 Spanish patients with psoriasis treated with biologics, members of Spanish Psoriasis Group, showed that 5.8%, 0.55%, and 0.45% of patients treated with efalizumab, infliximab, and etanercept, respectively developed PsA in 2010.

Different theories have been postulated in order to investigate the possible mechanism that may explain the onset of paradoxical reactions and therefore, new-onset PsA. Understanding the pathogenesis of paradoxical psoriasis may increase the knowledge about the onset of paradoxical PsA.

The most common theory is that TNF blockade may induce a dysregulated type I interferon (IFN-I) response and promote the formation of anti-nuclear antibodies [52]. In fact, paradoxical psoriasis developed during anti-TNFα therapy showed an increased IFN signature in affected skin [53]. Moreover, the production of I-IFN by plasmacytoid dendritic cells is controlled by TNF level; so anti-TNFα treatment causes an overexpression of I-IFN, which is linked to paradoxical psoriasis [52].

A similar pathogenetic pathway may be theorized in psoriatic patients treated with anti-TNFα who developed PsA; however, the onset of PsA during treatment with other biologics (anti-IL12/23, anti-IL17, and anti-IL23) and the absence of experimental evidence do not allow strongly supporting this theory currently.

Moreover, a review on paradoxical psoriasis developed during biologic therapies showed that classical and paradoxical psoriasis have clinical similarities but many pathophysiologic distinctions such as pathogenic mechanism (chronic autoimmune TH/TC17-mediated inflammation vs. possible type-I IFN-driven innate inflammation with the absence of T-cell autoimmunity), histopathological appearance (epidermal hyperplasia, papillomatosis, hyperparakeratosis, dermal, and epidermal immune cell infiltrates vs. classical psoriatic pattern and/or eczematous pattern and/or lichenoid pattern), and role of TNF (driven by TNF vs. induced by blockade of TNF) [52]. Thus, a pathophysiologic difference can be also assumed between PsA and paradoxical PsA. Certainly, further studies on molecular mechanisms underlying the pathogenesis of PsA and new-onset PsA are needed, potentially allowing to identify patients in whom a specific biologic drug may play a protective or risk factor.

The smoking paradox [54], which shows that smoking may have a protective role on the development of PsA in patients with psoriasis, further complicates an explanation that may clarify the relationship between PsA and psoriasis.

PsA development has been reported during biologic treatment for other diseases, too [55,56,57,58], such as rheumatoid arthritis and inflammatory bowel disease, advancing the idea that the possibility of the onset of PsA is not only connected to the disease treated with biologics, but it may be linked to the use of biologics themselves. However, it should be also stated that PsA is more frequent in patients with intestinal bowel diseases than in the general population [59].

The pathogenesis of PsA, including genetics, environmental factors, and immune-mediated inflammation complex, is poorly understood [60]. The use of biologics drugs for psoriasis may rarely cause a cytokine imbalance, which could sustain PsA development. Jones et al. [37] hypothesized that PsA may develop in patients treated with ustekinumab for lacking of efficacy at the given dosage. Čarija et al. [39] suggested that cytokine imbalance due to anti-TNF-α administration causing an overexpression of I-IFN and/or a lower dose of ustekinumab in patients with elevated body mass index resulting in lower efficacy may trigger or unmask PsA.

Our study highlights that new-onset PsA can occur during treatment with anti-TNFα, IL-23, IL-17, and 12/23, suggesting that there is not a specific cytokine blockage at the basis of PsA. The incidence of PsA in our study shows that the use of biologic drugs does not seem to completely protect against the possibility of a future development of PsA; hence, dermatologist attention and screening for PsA must continue also in patients under biologics. Conversely, it should be also underlined that biologic treatment strongly reduced the incidence of PsA, which can reach up to 20% of patients with psoriasis [61]. In fact, a recent 8-year retrospective non-randomized study [50] tried to assess the incidence of PsA in patients with moderate-to-severe plaque psoriasis comparing subjects under biologic treatment prescribed for at least 5 years (infliximab, etanercept, adalimumab, ustekinumab, secukinumab) with patients treated with narrow-band ultraviolet light B (nbUVB). A total of 464 subjects were divided in two groups: 234 were treated with biologics and 230 were treated with nbUVB with a mean follow-up period of 6.8 ± 1.4 years per person. An event per person-years analysis was used to assess the annual and cumulative incidence, which was 1.20 cases (95% CI 0.77 to 1.89) versus 2.17 cases (95% CI 1.53 to 3.06) per 100 patients/year in the biologics versus phototherapy group, respectively (HR 0.29, 0.12–0.70; *p* = 0.006). In particular, among patients treated with biologic drugs, 19/234 (8.1%) developed PsA vs. 32/230 (13.9%) in the nbUVB group. Moreover, an increased risk of PsA was found in patients with older age, psoriasis duration >10 years, and nail psoriasis. According to our study, Van Muijen et al. [23], and Napolitano et al. [22], the peripheral arthritis was the most frequent pattern of PsA (16/19, 84.2%).

The percentage of subjects who developed new-onset PsA during biologic treatment was similar to the one reported by our study (8.1% vs. 8.5%), reinforcing the idea that biologic therapy strongly reduces but does not completely eliminate the risk of PsA onset.

Acosta Felquer et al. [51] confirmed that the incidence of PsA in psoriatic patients treated with biologic drugs was significantly lower (incidence rate ratio (IRR) = 0.26; 95% CI 0.03 to 0.94; *p* = 0.0111) compared with patients treated with topical therapy, phototherapy, or no treatment. However, the authors also reported that PsA incidence under biolgics was not significantly lower compared with patients treated with conventional disease-modifying antirheumatic drugs (methotrexate and cyclosporine) (IRR = 0.35; 95% CI 0.035 to 1.96; *p* = 0.1007). Indeed, in their retrospective study, patients were divided in three groups: topics (1387, 81%: topics, phototherapy or no treatment), conventional disease-modifying antirheumatic drugs (229, 13%), and biological therapy (103, 6%: anti-TNF, anti-IL12/23, anti-IL1, and anti-IL23), and PsA was diagnosed during follow-up in 231 (16.7%), 6 (2.6), and 2 (1.95) patients, respectively.

Furthermore, the authors reported that male sex, nail involvement, and higher body mass index were associated with increased risk of developing PsA.

Reviewing the literature, characteristics that may strongly predict the development of PsA were not found, since there are very variable data. For example, the female prevalence reported by Van Muijen et al. [23] is supported also by our results but not by Napolitano et al. The correlation with body mass index suggested by Asahina et al. [24] and the correlation with PASI showed by Napolitano et al. [22] were not confirmed in the other studies.

Trying to understand the pathogenetic mechanisms of new-onset PsA during biologic treatment is essential to find predictive factors that can help physicians distinguish patients with high or low risk of PsA development. In our opinion, new-onset PsA and classical PsA may be two different entities with similar clinical manifestations that hide different pathogenetic mechanisms. Furthermore, PsA may develop in parallel with psoriasis, and biologic drugs did not protect against its development.

## 5. Limitation of the Study

The relatively small sample size of this prospective study is the major limitation. Additional limitations could be represented by the features of a small percentage of follow-up visits. Even if the COVID-19 pandemic period changed our routine work, with most of the visits (533/590, 90.3%) being conducted in attendance and respecting all the precautionary measures, the remaining were performed via telemedicine (57/590, 9.7%). Even if all the baseline visits were conducted in attendance and telemedicine was done only in 9.7% of the total visits, we cannot definitively exclude that this would not have influenced the final data. Moreover, the comparison between the drugs could have been influenced by the low number of patients treated with anti-IL23.

Finally, 3/118 patients (2.5%) spontaneously and independently suspended biologic therapy for 1.3 ± 0.6 months during the follow-up period due to the fear of Sars-CoV2 infection.

## 6. Conclusions

Data on new-onset PsA in patients with psoriasis treated with biologic drugs are very limited, showing variable and conflicting results.

Our study revealed that 8.5% (10 out of 118) psoriasis patients under biologics developed PsA during one-year follow-up, showing that new-onset PsA is possible for each existing class of biologics (anti-TNF, anti-IL12/23, anti-IL1, and anti-IL23), without any drug predilection and with the peripheral form representing most of the cases. Without any doubt, treating psoriasis with biologic drugs reduces the risk of PsA development [50,51].

However, clinicians must keep in mind the possibility of new-onset PsA onset also in patients undergoing biologic treatment so that PsA screening should be strongly recommended for each follow-up visit. Further studies are needed to clarify the pathogenesis of PsA, eventual risk factors, and deepen the correlation between biologic therapy and new-onset PsA in order to allow finding predictive factors that could help in preventing these events and choose the best tailored-tail therapy for each patient. Particularly, investigations on genetic polymorphism, microRNAs, serum biomarkers, and combined data analysis with artificial intelligence may guide future studies in order to identify specific PsA biomarkers, thus facilitating the study of new onset PsA.

## Figures and Tables

**Table 1:** Study population, patients who developed or did not develop new-onset PsA during biologic treatment: general features.

	Study Population	PsA Development	Non PsA	P
**Number of patients**	118	10 (8.5%)	108 (91.5%)	
**Sex:**				
Male	76 (64.4%)	3 (30.0%)	73 (67.6%)	ns
Female	42 (35.6%)	7 (70.0%)	35 (32.4%)	ns
**Mean age** *(years)*	48.4 ± 14.2	44.5 ± 13.4	48.8 ± 14.2	ns
**Mean duration of Psoriasis** *(years)*	18.4 ± 12.4	14.2 ± 8.3	18.8 ± 12.6	ns
**Psoriasis activity**				
PASI	2.2 ± 6.2	1.8 ± 2.9	2.2 ± 6.5	ns
BSA	2.6 ± 5.6	4.6 ± 8.7	2.4 ± 5.2	ns
**Time of treatment** *(months)*	36.1 ± 26.4	26.1 ± 21.6	37.0 ± 26.7	ns
**Comorbidities:**				
Absence of comorbidities	59 (50.0%)	6 (60.0%)	53 (49.1%)	ns
Hypertension	16 (13.6%)	1 (10.0%)	15 (13.9%)	ns
Diabetes mellitus	12 (10.2%)	1 (10.0%)	11 (10.2%)	ns
Dyslipidemia	24 (20.3%)	1 (10.0%)	23 (21.3%)	ns
Hidradenitis	1 (0.8%)	0	1 (0.9%)	ns
Obesity	15 (12.7%)	0	15 (13.9%)	ns
Hypothyroidism	2 (1.7%)	1 (10.0%)	1 (0.9%)	ns
Hyperthyroidism	0	0	0	ns
Acute myocardial infarction	1 (0.8%)	0	1 (0.9%)	ns
Cardiopathy	2 (1.7%)	0	2 (1.9%)	ns
Chronic obstructive pulmonary disease	2 (1.7%)	0	2 (1.9%)	ns
Hepatitis B	1 (0.8%)	0	1 (0.9%)	ns
Hepatitis C	1 (0.8%)	0	1 (0.9%)	ns
Polycistic ovary syndrome	1 (0.8%)	0	1 (0.9%)	ns
Other (vitiligo, glaucoma, ulcerative colitis, hives)	5 (4.2%)	1 (10.0%)	4 (3.7%)	ns
**Previous treatments (conventional and small-molecules):**				
Apremilast	5 (4.2%)	1 (10.0%)	4 (3.7%)	ns
Methotrexate	40 (33.9%)	3 (30.0%)	37 (34.3%)	ns
Cyclosporine	47 (39.8%)	2 (20.0%)	45 (41.7%)	ns
Acitretin	10 (8.5%)	1 (10.0%)	9 (8.3%)	ns
Topical corticosteroids	49 (41.5%)	6 (60.0%)	43 (39.8%)	ns
Nb-UVB Phototherapy	13 (11.0%)	1 (10.0%)	12 (11.1%)	ns
Dimethyl-fumarate	1 (0.8%)	0	1 (0.9%)	ns
Psoralen-UVA	2 (1.7%)	0	2 (1.9%)	ns
**Previous biologic treatments:**				
Biologic naïve	84 (71.2%)	5 (50%)	79 (73.1%)	ns
**Anti-TNF-α**	26 (22.0%)	4 (40.0%)	22 (20.4%)	ns
Anti IL-23	1 (0.8%)	1 (10.0%)	0	ns
Anti IL-17	14 (11.9%)	2 (20.0%)	12 (11.1%)	ns
Anti IL-12/23	9 (7.6%)	2 (20.0%)	7 (6.5%)	ns
**Current biologic treatment:**				
**Anti-TNFα**				
Etanercept	4 (3.4%)	1 (10.0%)	3 (2.8%) (75.0%)	ns
Adalimumab	25 (21.2%)	2 (20.0%)	23 (21.3%) (92.0%)	ns
Certolizumab	2 (1.7%)	0	2 (1.9%) (100%)	ns
**Anti-IL12/23**				
Ustekinumab	27 (22.9%)	2 (20.0%)	25 (23.1%) (92.6%)	ns
**Anti-IL17**				
Secukinumab	13 (11.0%)	2 (20.0%)	11 (33.3%) (84.6%)	ns
Ixekizumab	25 (21.2%)	1 (10.0%)	24 (22.2%) (96.0%)	ns
Brodalumab	6 (5.1%)	1 (10.0%)	5 (4.6%) (83.3%)	ns
**Anti-IL23**				
Risankizumab	1 (0.9%)	1 (10.0%)	0	ns
Guselkumab	11 (9.3%)	0	11 (10.2%) (100%)	ns
Tildrakizumab	4 (3.4%)	0	4 (3.7%) (100%)	ns

ns stands for non-significant.

**Table 2:** Patients who developed new-onset PsA during biologic treatment: general features, treatment, and PsA severity. MTX: methotrexate, SSZ: sulfasalazine, CS: oral corticosteroids.

Patient	Sex	Age	Psoriasis Duration	Biologic Treatment	Treatment Duration	Therapy Following the Diagnosis of PsA	PsA Severity
1	Female	35	17	Adalimumab	12	Ixekizumab	Moderate
2	Male	52	15	Secukinumab	32	Secukinumab + MTX	Moderate
3	Female	30	10	Ustekinumab	60	Ustekinumab + CS	Mild
4	Female	46	3	Adalimumab	18	Secukinumab	Severe
5	Female	50	30	Etanercept	56	Certolizumab	Severe
6	Female	27	7	Ustekinumab	18	Ustekinumab + SSZ	Mild
7	Female	38	21	Brodalumab	3	Brodalumab + MTX	Moderate
8	Male	73	5	Ixekizumab	12	Ixekizumab + MTX	Moderate
9	Male	42	14	Secukinumab	48	Secukinumab + CS	Mild
10	Female	52	20	Risankizumab	2	Risankizumab + MTX	Mild

**Table 3:** Studies reporting the development of new-onset PsA in patients with moderate-to-severe psoriasis treated with biologic therapy. * PASI at the diagnosis of PsA. ** Period of study enrolment.

Reference	Type of Study	Treatment	New-Onset PsA	Male	Age (Years)	PASI *	Period **
Napolitano et al. [22]	Retrospective	Anti-TNFα, Anti-IL12/23	22/327 (6.7%)	15/22 (68.2%)	51.4 ±9.1	18.7 ± 12.1	2011–2015
Van Muijen et al. [23]	Prospective	Anti-TNFα, Anti-IL12/23, Anti-IL17	32/342 (9.4%)	15/32 (46.9%)	57.2 ± 14.1	6.6 ± 6.6	2005–2018
Asahina et al. [24]	Retrospective	Anti IL12/23	8/179 (4.5%)	6/8 (75%)	64.1 ± 18.8	12.0 ± 5.1	2011–2015
Takahashi et al. [35]	Case report	Anti-IL12/23	1	1 (100%)	79	1.9	-----------------
De Souza et al. [36]	Case series	Anti-IL12/23	2	2 (100%)	39.5 ± 2.1	3.9 ± 1.8	-----------------
Jones et al. [37]	Case series	Anti-IL12/23	5	0 (0%)	59 ± 8	-----------------	-----------------
Stamell et al. [38]	Case series	Anti-IL12/23	2	2 (100%)	45 ± 7.1	-----------------	-----------------
Carija et al. [39]	Case report	Anti-IL 12/23	1	1 (100%)	46	-----------------	-----------------
Di Costanzo et al. [40]	Case series	Anti-TNFα	1	1 (100%)	46	-----------------	-----------------
Vidal et al. [41]	Case report	Anti IL17	1	1 (100%)	46	10	-----------------
Gisondi et al. [50]	Retrospective	Anti-TNFα, Anti-12/23, Anti-IL17	19/234 (8.1%)	-----------------	-----------------	-----------------	2012–2020
Acosta Felquer et al. [51]	Retrospective	Anti-TNFα, Anti-IL12/23, Anti-IL17	2/103 (1.9%)	67/103 (65.0%)	-----------------	-----------------	2000–2018

----------------- stands for “not reported”.

## Data Availability

All collected data are reported in the study.
